# Potential Therapeutic Benefits of Pepper Fruit Extract on Hyperglycemia and Dyslipidemia in Obese Type 2 Diabetic Mice

**DOI:** 10.7759/cureus.89199

**Published:** 2025-08-01

**Authors:** Morteza Sadeghi, Mahvash Afshari, Jin Pyo Oh, Tae Ho Ryu, Hye Ran Choi, Ha Na Jang, Gi Jun Kim, Sanghyeob Lee

**Affiliations:** 1 Biochemistry, Sa.C. Islamic Azad University, Sanandaj, IRN; 2 Bioresource Engineering, Sejong University, Seoul, KOR; 3 Biotechnology and Breeding, Asia Seed Co., Icheon, KOR; 4 Food and Nutrition, Gochang Food and Industry Institute, Gochang, KOR

**Keywords:** db/db mouse model, glucose regulation, insulin resistance, pepper fruit extract, type 2 diabetes

## Abstract

Background

Type 2 diabetes (T2D) is a complex metabolic disorder characterized by impaired glucose regulation and insulin resistance and frequently accompanied by obesity and dyslipidemia. The search for novel therapeutic agents to manage these metabolic parameters remains ongoing. Pepper fruit (cv. *Meein*) extract, a non-pungent *Capsicum* variant, has been proposed as a potential intervention for metabolic dysfunctions associated with T2D.

Methods

This study investigated the impact of pepper fruit extract on metabolic parameters using a *db/db* diabetic mouse model. Fifty mice were randomly assigned to five groups (n = 10 per group): a normal control group, a diabetic control group (*db/db*), a positive control group treated with acarbose (50 mg/kg), and two treatment groups receiving either 150 mg/kg or 300 mg/kg of "*Meein*" extract. The intervention lasted for 10 weeks. Key metabolic indicators, including body weight, blood glucose levels, oral glucose tolerance (OGTT), lipid profile (triglycerides and cholesterol), and insulin concentrations, are measured to assess the extract's therapeutic potential.

Results

Treatment with "*Meein*" extract resulted in significant reductions in body weight, with the 300 mg/kg group exhibiting a 10.6% decrease compared to the diabetic control. Both treatment groups demonstrated reductions in blood glucose, with the 150 mg/kg group showing statistically significant improvements. OGTT results did not indicate significant enhancement in glucose tolerance, and lipid profiles, including triglyceride and cholesterol levels, did not show significant changes following extract administration. Notably, the extract exerted a marked effect on insulin levels: while the diabetic control group showed hyperinsulinemia, insulin concentrations in the extract-treated groups were significantly reduced, with the 300 mg/kg group achieving a 38% decrease, indicative of improved insulin sensitivity.

Conclusion

These findings suggest that ‘*Meein*’ extract may have potential for managing obesity and improving insulin sensitivity in T2D. Importantly, no significant effects on OGTT or lipid profiles were observed in this study. Further research is necessary to clarify the extract’s effects on glucose metabolism and lipid regulation.

## Introduction

Diabetes mellitus is a chronic metabolic disorder characterized by persistent hyperglycemia due to impaired insulin secretion, action, or both [1-3]. This disruption in glucose metabolism results from either insufficient insulin production or ineffective insulin utilization [4-6]. In addition to hyperglycemia, hyperlipidemia, and oxidative stress are major contributors to disease progression. Elevated cholesterol and triglycerides increase cardiovascular risk, while oxidative stress exacerbates beta-cell dysfunction and inflammation [7-11].

With diabetes on the rise globally, the disease poses a significant public health challenge. The high cost and side effects of current treatments have spurred interest in preventive strategies, especially among pre-diabetic individuals [12-14]. This has led to growing interest in natural compounds with anti-diabetic properties.

Peppers (*Capsicum spp.*) are widely consumed and valued not only for flavor but also for their medicinal properties [15]. Capsaicin, the primary capsaicinoid, has been widely studied for its effects on glucose metabolism [16,17]. Other pepper-derived compounds, including flavonoids, carotenoids, and vitamins, also contribute to blood sugar regulation and improved insulin sensitivity [18-20].

The non-pungent “*Meein*” pepper cultivar is compositionally and functionally distinct from other studied pepper cultivars, primarily due to its lack of capsaicinoids, the compounds responsible for the characteristic pungency or spiciness of peppers such as capsaicin and dihydrocapsaicin. While most traditional pepper cultivars, like *C. annuum* or *C. frutescens*, contain varying levels of these pungent compounds, the “*Meein*” cultivar has either undetectable or very low levels of capsaicinoids. Preliminary in vitro studies suggest that the non-pungent “*Meein*” pepper cultivar may possess anti-diabetic effects [21]. This study investigates its impact on glycemic control, insulin, and lipid profiles in a diabetic rat model and compares its efficacy to acarbose, a standard anti-diabetic drug.

## Materials and methods

Preparation of “*Meein*” extracts

Sixty kilograms of unripe “*Meein*” green pepper fruits were stemmed, deseeded, and dried for two days using a pepper dryer (Green Farm, Daegu, Korea). The dried fruit was ground into 3 kg of powder and extracted in hot water for 16 h at the Berry and Bio Food Research Institute (Gochang, Korea). The extract was vacuum-dried to yield 500 g of powder. For use, 30 g of this powder was mixed with 240 mL of water, incubated at 55°C for 16 h, filtered (PD-10 column, GE Healthcare, IL, USA), and concentrated (CVE-2200, Eyela, Japan). Final extracts were stored at 4°C.

Animal care and experimental design

Eight-week-old male *db/db* diabetic mice and non-diabetic C57BL/6N controls were obtained from Harlan Sprague Dawley Inc. (IN, USA). After a one-week acclimation period, mice were randomly divided into five groups (n = 10 per group) based on body weight and fasting glucose levels. All mice were housed under controlled environmental conditions (22 ± 2°C, 50 ± 5% humidity, 12-h light/dark cycle) and provided a high-fat diet (Purina, Korea). The experimental groups included: normal control (C57BL/6), diabetic control (*db/db*), *db/db* + 150 mg/kg “*Meein*” extract, *db/db* + 300 mg/kg “*Meein*” extract, and *db/db* + 50 mg/kg acarbose (Sigma-Aldrich, MO, USA). Treatments were administered orally by gavage once daily at 10:00 AM for 10 weeks. Control groups received an equivalent volume of phosphate-buffered saline (PBS) (Table 1). All procedures were approved by the Institutional Animal Ethics Committee of Berry and Bio Food Research Institute Efficacy Evaluation Center (BBRI-IACUC-23010).

**Table 1:** Experimental groups. PBS: phosphate-buffered saline

Group	Experimental animals	Substance administered	N
Control (Normal)	C57BL/6	PBS	10
Control (Diabetic)	*db/db* mouse	PBS	10
Control (Positive)	*db/db* mouse	Acarbose 50 mg/kg	10
Low-dose	*db/db* mouse	“*Meein*” fruit extract 150 mg/kg	10
High-dose	*db/db* mouse	“*Meein*” fruit extract 300 mg/kg	10

Data collection and biochemical parameter assessments

Body weight and fasting blood glucose (after a 15 h fast) were measured weekly using a digital scale and a glucometer (Accu-Chek Performa, Roche, Switzerland) via tail vein sampling [22]. For the oral glucose tolerance (OGTT), mice received an oral dose of glucose (2 g/kg in 0.2 mL PBS) after fasting, and blood glucose levels were recorded at zero, 30, 60, and 120 min. At the end of the 10-week study, mice were again fasted for 15 h, anesthetized with isoflurane, and blood was collected from the abdominal aorta. Plasma was separated by centrifugation (2,000 ×g, 10 min) for analysis of triglycerides and total cholesterol. Plasma insulin levels were quantified using an enzyme-linked immunosorbent assay (ELISA) kit (Crystal Chem, IL, USA), with absorbance measured at 450 nm (Multiskan FC, Thermo Fisher Scientific, MA, USA) [23]. Insulin resistance was calculated below.

$\text{Blood Insulin Level} = \frac{\text{Fasting Insulin} \times \text{Fasting Glucose}}{405}$

Statistical analysis

Normality of the data was assessed using the Kolmogorov-Smirnov test in SAS version 9.4 (SAS Inc., Cary, NC, USA, https://www.sas.com/). For quantitative comparisons, independent two-sample t-tests were conducted to evaluate differences between each treatment group and the diabetic control group (*db/db*). Statistical significance is denoted by p < 0.05, p < 0.01, and p < 0.001 relative to the diabetic control. Data are presented as mean ± standard error of the mean (SEM). All graphical representations were created using GraphPad Prism version 9.0 (Dotmatics, Boston, MA, USA, https://www.graphpad.com/).

## Results

Effect on body weight

**Figure 1:**
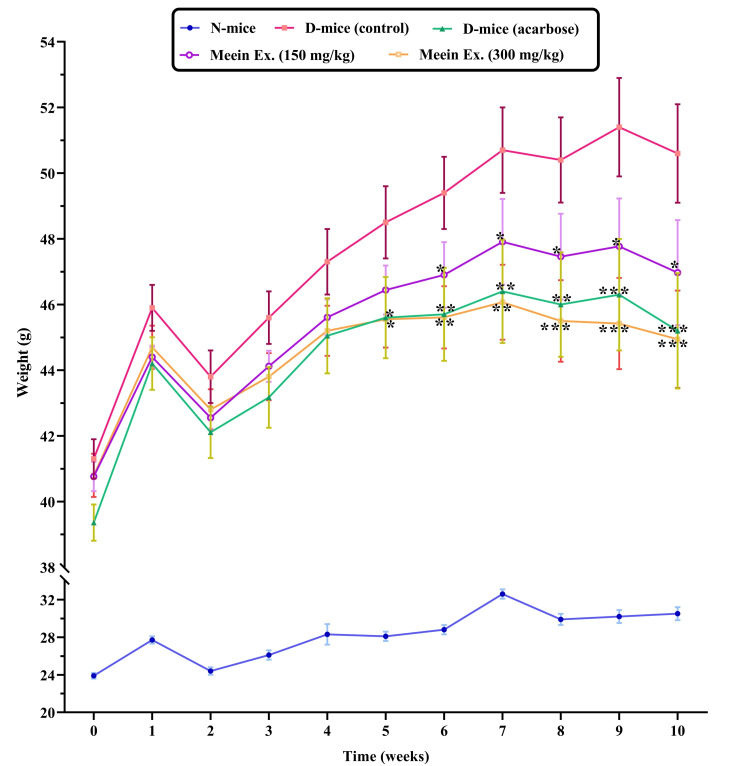
Body weights measured over a 10-week period. N: normal group; D: diabetic group; Ex: extract Statistical significance, assessed using t-test analysis, is indicated by *p < 0.05, **p < 0.01, and ***p < 0.001 compared to the diabetic control group (n = 10 per group).

Over the 10-week treatment period, significant differences in body weight were observed among groups (Figure* *1). The untreated diabetic control group (*db/db*) showed a 39.8% increase in body weight compared to the normal control, consistent with diabetes-related metabolic dysfunction. In contrast, mice treated with 150 mg/kg of "*Meein*" fruit extract exhibited a 7.9% reduction in body weight by the end of the study, becoming noticeable after three weeks. The 300 mg/kg group demonstrated an even greater reduction of 10.6%, apparent between weeks three to seven (p < 0.01). This effect was comparable to the acarbose-treated group, suggesting that "*Meein*" extract-particularly at higher doses-may help manage diabetes-induced weight gain.

Fasting blood glucose levels

**Figure 2:**
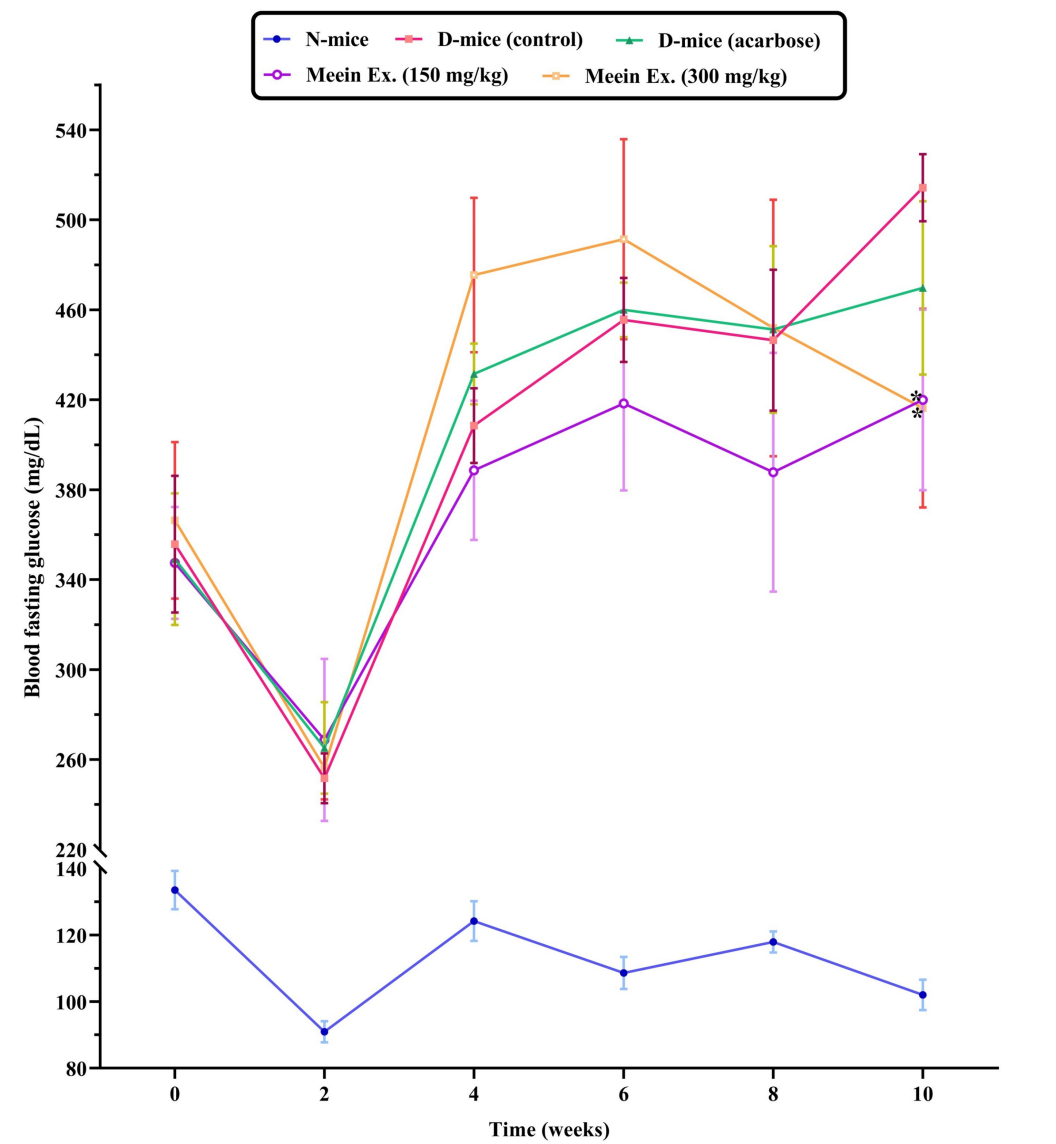
Fasting blood glucose levels recorded over a 10-week period. N: normal group; D: diabetic group; Ex: extract Statistical significance, assessed using t-test analysis, is indicated by *p < 0.05 compared to the diabetic control group (n = 10 per group).

At baseline, diabetic mice had significantly higher fasting blood glucose (363.1 ± 33.0 mg/dL) than the normal control group (133.5 ± 5.7 mg/dL). After 10 weeks, levels increased further in the diabetic control group (514.3 ± 14.9 mg/dL), indicating worsening hyperglycemia. Treatment with 150 mg/kg and 300 mg/kg "*Meein*" extract lowered glucose levels to 419.9 ± 40.2 and 416.3 ± 44.2 mg/dL, respectively (Figure 2). The 150 mg/kg dose showed a statistically significant reduction compared to the diabetic control (p < 0.05). Acarbose treatment also reduced glucose (469.7 ± 38.6 mg/dL), though not significantly.

Oral glucose tolerance (OGTT)

**Figure 3:**
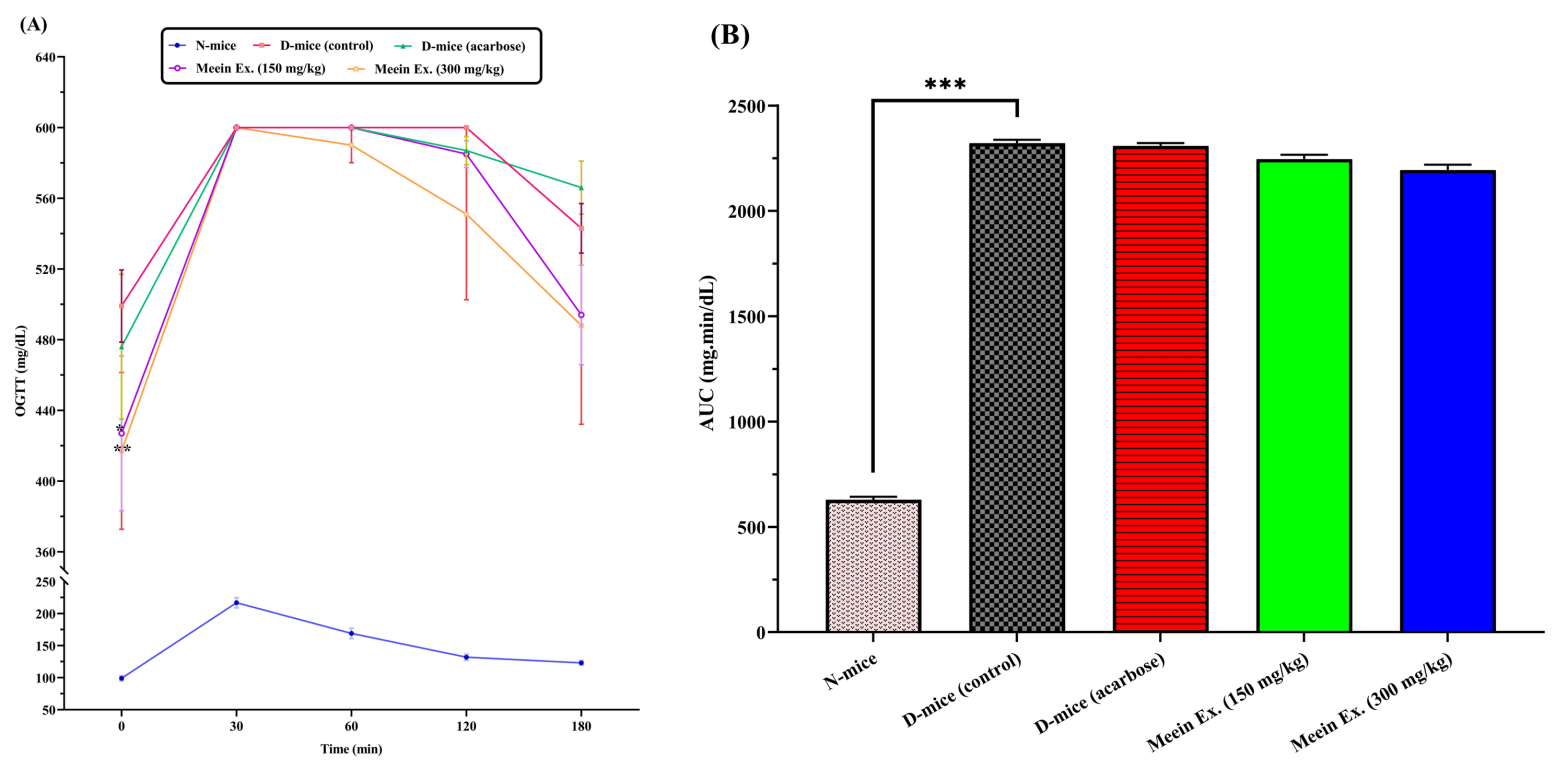
Oral glucose tolerance test (OGTT) and area under the curve (AUC) analysis. A: OGTT results and B: corresponding AUC. N: normal group; D: diabetic group; Ex: extract The AUC was calculated for the entire 180-minute duration of the OGTT, representing cumulative glucose levels over this period. Statistical significance, assessed using t-test analysis, is indicated by *p < 0.05, **p < 0.01, and ***p < 0.001 compared to the diabetic control group (n = 10 per group).

Glucose tolerance was assessed via OGTT (Figure 3). Peak glucose levels were observed between 30 and 60 min post-administration in all groups. While no statistically significant improvements were found in the extract-treated groups, area under the curve (AUC) analysis showed a modest reduction in glucose levels: 2,245.5 ± 20.9 mg·min/dL (150 mg/kg), 2,193.5 ± 20.6 mg·min/dL (300 mg/kg), versus 2,321 ± 12.3 mg·min/dL in the diabetic control (p < 0.001). The 300 mg/kg *Meein* extract group exhibited the lowest AUC (2,193.5 ± 20.6 mg·min/dL), indicating a trend toward improved glucose regulation compared to both the diabetic control and the acarbose group (2,308 ± 13.9 mg·min/dL), although these differences were not statistically significant.

Lipid profile: triglycerides and cholesterol

**Figure 4:**
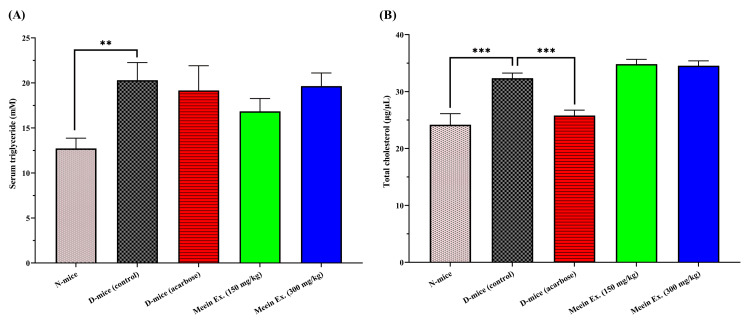
Biochemical analysis. A: serum triglycerides and B: total cholesterol. N: normal group; D: diabetic group; Ex: extract Serum triglyceride and total cholesterol levels were measured after a 15-hour fasting period at the end of the 10-week experimental period. Statistical significance, assessed using *t*-test analysis, is indicated by **p < 0.01 and ***p < 0.001 compared to the diabetic control group (n = 10 per group).

Triglyceride levels were elevated in the diabetic control group (20.3 ± 2.0 mM), about 37.3% higher than in normal controls (16.8-19.2 mM) (Figure 4). Treatment with "*Meein*" extract did not significantly reduce triglyceride levels at either dose. Total cholesterol levels in the diabetic control group (24.2-32.3 μg/μL) were approximately 20% lower than in the normal control group (25.8-34.8 μg/μL), contrary to the typical pattern expected in diabetic dyslipidemia (p < 0.001). Treatment with ‘*Meein*’ extract did not result in significant changes in cholesterol levels at either dose.

Insulin levels and resistance

**Figure 5:**
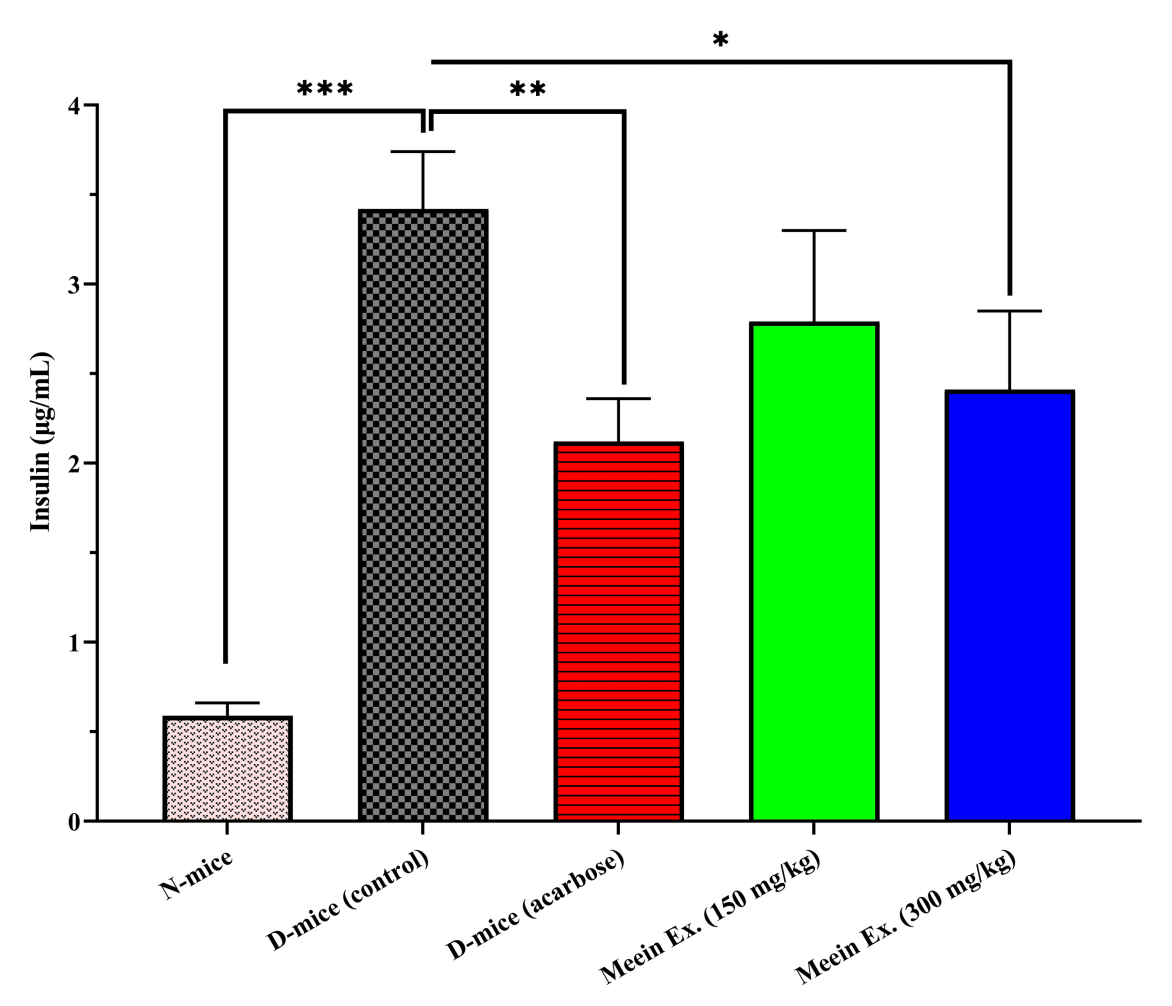
Insulin level measurement. N: normal group; D: diabetic group; Ex: extract Insulin level was measured after a 15-hour fasting period at the end of the 10-week experimental period. Statistical significance, assessed usingt-test analysis, is indicated by *p < 0.05, **p < 0.01, and ***p < 0.001 compared to the diabetic control group (n = 10 per group).

Insulin concentrations were markedly higher in diabetic mice (3.42 ± 0.32 µg/mL) compared to the normal control (0.59 ± 0.07 µg/mL) (p < 0.001), indicating compensatory hyperinsulinemia (Figure 5). "*Meein*" extract significantly reduced insulin levels to 2.12 ± 0.24 µg/mL (150 mg/kg) and 2.79 ± 0.51 µg/mL (300 mg/kg), with the 300 mg/kg group showing a ~38% reduction from diabetic controls. Acarbose treatment also lowered insulin to 2.11 ± 0.24 µg/mL. These findings suggest that "*Meein*" extract may improve insulin sensitivity, similar to the effects of acarbose.

## Discussion

Insulin is a critical peptide hormone produced by pancreatic β-cells that regulates glucose homeostasis by facilitating glucose uptake in insulin-sensitive tissues such as muscle, liver, and adipose tissue. In type 2 diabetes (T2D), chronic insulin resistance disrupts this regulatory system, often resulting in elevated blood glucose levels and compensatory hyperinsulinemia as pancreatic β-cells attempt to maintain normoglycemia [24]. In the present study, significantly higher insulin levels were observed in the diabetic control group, reflecting the typical pathophysiological hallmark of T2D. These findings are consistent with prior studies using *db/db* mice as a model for insulin resistance and β-cell dysfunction [25].

Interestingly, administration of “*Meein*” extract led to a substantial and statistically significant reduction in circulating insulin levels, especially in the group treated with 300 mg/kg extract, which showed a 38% decrease compared to the diabetic control. This observation suggests that the extract may enhance insulin sensitivity or reduce the pancreatic β-cell burden by improving peripheral glucose utilization. Previous studies suggest that bioactive compounds in “*Meein*” may act through modulation of TRPV1 receptors and downstream signaling pathways that enhance insulin sensitivity and promote glucose uptake [26]. Lower insulin levels under hyperglycemic conditions may indicate more efficient glucose clearance without the need for excessive insulin secretion, a desirable outcome in the management of T2D [27].

Restoration of insulin sensitivity not only improves glycemic control but may also reduce the risk of chronic complications associated with persistent hyperinsulinemia, including cardiovascular disease, obesity, and metabolic syndrome [28]. Therefore, the observed decrease in insulin levels following treatment with “*Meein*” extract could have broader implications for long-term diabetes management and metabolic health.

In parallel, changes in body weight among the experimental groups provide further support for the extract’s metabolic effects. *db/db* mice typically exhibit significant weight gain due to leptin receptor deficiency, hyperphagia, and insulin resistance [29]. Consistent with this, the diabetic control group gained significant weight over the experimental period. In contrast, mice treated with “*Meein*” extract, particularly at 150 and 300 mg/kg, exhibited reduced weight gain, with notable differences emerging from the third week of treatment. This early onset of weight modulation suggests that the extract acts rapidly, potentially through mechanisms involving increased energy expenditure, appetite regulation, or improved lipid metabolism [19]. Weight reduction is particularly important in T2D, as sustained weight loss is closely linked with enhanced insulin sensitivity, improved glycemic control, and reduced comorbidities [30]. The observed effects imply that “*Meein*” extract may target multiple metabolic pathways, offering both glucose-lowering and anti-obesity benefits.

The “*Meein*” extract’s hypoglycemic potential was further supported by fasting blood glucose measurements. Diabetic control mice exhibited markedly elevated glucose levels, characteristic of *db/db* mice with severe insulin resistance and hyperphagia due to leptin dysfunction [31]. Treatment with 150 mg/kg “*Meein*” extract significantly reduced glucose levels from 514.3 ± 14.9 mg/dL to 419.9 ± 40.2 mg/dL. Although the 300 mg/kg dose did not outperform the lower dose, this may suggest a biphasic or non-linear dose-response relationship, where excessive dosing might reduce efficacy or activate counter-regulatory mechanisms. Such dose-dependent variation has been observed in plant-based interventions and warrants further investigation to optimize therapeutic outcomes.

Notably, the acarbose-treated group showed only modest improvement, with no significant reduction in blood glucose. Acarbose is an α-glucosidase inhibitor that slows carbohydrate absorption but does not directly enhance insulin action or glucose uptake [32]. The superior performance of “*Meein*” extract at the 150 mg/kg dose implies a broader mode of action, possibly involving both insulin signaling and hepatic glucose regulation. These findings position the extract as a potentially more comprehensive intervention for glycemic control compared to enzyme inhibitors alone.

OGTT results provided additional insights. All groups demonstrated peak glucose levels between 30 and 60 min post-glucose administration, reflecting normal physiological kinetics [33]. While the glucose curves of extract-treated groups did not significantly differ from the diabetic control, analysis of the AUC revealed meaningful trends. Lower AUC values in the 150 and 300 mg/kg extract groups, as well as in the acarbose group, suggest improved glucose regulation over time. A reduced AUC indicates faster glucose clearance and better postprandial regulation, which are key for minimizing chronic hyperglycemia-associated damage such as oxidative stress, endothelial dysfunction, and microvascular complications [34].

Although the reductions in AUC did not achieve statistical significance, they were clinically relevant and suggest that longer treatment duration or a larger sample size may yield stronger effects. Furthermore, the extract may require chronic administration to exert full benefits on glucose tolerance, as acute testing may underestimate the cumulative metabolic adaptations induced by plant-derived bioactives. These observations call for extended-duration studies with repeated OGTT assessments to confirm efficacy.

The extract’s effects on lipid profiles were also assessed, considering that dyslipidemia frequently coexists with T2D. Hypertriglyceridemia is particularly common and has been linked not only to cardiovascular disease but also to diabetic nephropathy, neuropathy, and hepatic steatosis [35]. Although administration of “*Meein*” extract reduced triglyceride and total cholesterol levels, the reductions were not statistically significant. Similarly, acarbose did not significantly affect triglyceride levels but did produce a notable reduction in total cholesterol (p < 0.001). These findings suggest that while the extract shows potential for improving glycemic indices, its effects on lipid metabolism may be less pronounced or require longer intervention. Future studies should explore whether synergistic combinations with lipid-lowering agents or optimization of extract composition might amplify these benefits.

Although the extract demonstrated statistically significant effects on fasting glucose and weight control, improvements in glucose tolerance and lipid parameters were less robust and did not reach statistical significance. The insulin-lowering effect suggests enhanced insulin sensitivity. These outcomes are comparable to those of acarbose and support the potential of *Meein *extract as an adjunct in diabetes management (Table 2).

**Table 2:** Comparison of Meein extract and control sample with previous studies. AUC: area under the curve; ns: not significant; OGTT: oral glucose tolerance test

Parameter	Normal control	Diabetic control (*db/db*)	*Meein* 150 mg/kg	*Meein* 300 mg/kg	Acarbose	Comparable published extracts (with ref.)
Body weight (%)	-	39.8	–7.9	–10.6	Comparable to 300 mg/kg	Mulberry extract: –8% [36]; Green tea: –13% [37]
Fasting blood glucose (mg/dL)	133.5 ± 5.7	514.3 ± 14.9	419.9 ± 40.2	416.3 ± 44.2	469.7 ± 38.6	Mulberry extract: 402 [36]; Berberine: 350 [38]
OGTT AUC (mg·min/dL)	-	2,321 ± 12.3	2,245.5 ± 20.9	2,193.5 ± 20.6	2,308 ± 13.9	Baicalin: 2,150 [39]
Triglycerides (mM)	16.8–19.2	20.3 ± 2.0	ns	ns	ns	Mulberry extract: ↓12% [36]
Cholesterol (μg/μL)	25.8–34.8	24.2–32.3	ns	ns	ns	Berberine: ↓20% [38]
Insulin (μg/mL)	0.59 ± 0.07	3.42 ± 0.32	2.12 ± 0.24	2.79 ± 0.51	2.11 ± 0.24	Green tea: –1.14± 0.22 [37]

Taken together, the results of this study highlight the multifaceted metabolic benefits of “*Meein*” fruit extract in a T2D mouse model. The extract demonstrated significant effects on insulin levels, fasting glucose, and body weight, and it showed promising trends in glucose tolerance and lipid modulation. These findings suggest that the extract acts through a combination of mechanisms, potentially involving enhanced insulin sensitivity, improved glucose uptake, modulation of hepatic glucose production, and mild effects on lipid metabolism.

Limitations

Several limitations should be acknowledged to provide a balanced interpretation of the data and to guide future research. First, although the 10-week intervention period was sufficient to reveal early metabolic improvements, it may not capture the long-term efficacy or safety of the "*Meein*" extract. Chronic administration studies are needed to assess sustained outcomes and identify any delayed adverse effects. Second, the study focused primarily on metabolic endpoints without exploring the underlying molecular mechanisms. Elucidating the signaling pathways involved in glucose and lipid regulation will be essential to fully understand the extract’s mode of action. Third, the exclusive use of male*db/db* mice limits the generalizability of the results, as sex-based differences in metabolic responses are well documented. Future studies should incorporate both sexes and alternative animal models to strengthen external validity. Lastly, although two doses were tested, the absence of a full dose-response analysis restricts conclusions regarding the optimal therapeutic range. A broader range of dosages should be evaluated in future studies to refine dosing strategies and ensure safety.

To address these limitations, we intend to conduct comprehensive toxicity and biocompatibility evaluations in subsequent studies, complemented by detailed histopathological examinations of major organs. Furthermore, we plan to utilize advanced molecular techniques, including ELISA for the quantification of proinflammatory cytokines, as well as quantitative polymerase chain reaction (qPCR) and Western blot analyses, to elucidate the molecular mechanisms underlying the effects of “*Meein*” extract on obesity and T2D. These methodological enhancements are expected to provide more profound insights into the safety profile, therapeutic efficacy, and involved molecular pathways of the extract, thereby ensuring that our findings are robust, reproducible, and mechanistically well-substantiated.

## Conclusions

In summary, “*Meein*” fruit extract showed significant benefits in a T2D mouse model by reducing insulin levels, lowering fasting blood glucose, and limiting diabetes-related weight gain, indicating improved insulin sensitivity and glycemic control. Although improvements in glucose tolerance and lipid profiles were less pronounced, the extract’s multi-mechanistic effects are promising. The present findings indicate that '*Meein*' extract demonstrates therapeutic promise in obesity management and enhancement of insulin sensitivity in T2D. Notably, this investigation revealed no statistically significant alterations in OGTT results or lipid profile parameters. Additional research is warranted to elucidate the extract's mechanisms of action regarding glucose homeostasis and lipid metabolism. Further studies are needed to isolate active compounds, clarify molecular mechanisms, optimize dosing, and confirm long-term safety and efficacy through animal and human trials. Exploring combinations with current therapies could also enhance its potential as a natural treatment for T2D and related metabolic disorders.
